# Seasonal and inter‐seasonal RSV activity in the European Region during the COVID‐19 pandemic from autumn 2020 to summer 2022

**DOI:** 10.1111/irv.13219

**Published:** 2023-11-20

**Authors:** Margaux M. I. Meslé, Mary Sinnathamby, Piers Mook, The WHO European Region Respiratory Network Group, Richard Pebody, Anissa Lakhani, Maria Zambon, Odette Popovici, Mihaela Lazăr, Amela Dedeić Ljubović, Nina Rodić Vukmir, Ayşe Başak Altaş, Emine Avci, Katarzyna Łuniewska, Karol Szymański, Greta Gargasiene, Svajune Muralyte, Ausra Dziugyte, Graziella Zahra, Ana Rita Gonçalves, Tania Spedaliero, Guillaume Fournier, Daniel Alvarez‐Vaca, Goranka Petrović, Irena Tabain, Katarina Prosenc, Maja Socan, Jelena Protic, Dragana Dimitrijevic, Alina Druc, Mariana Apostol, Kate Karolina Kalasnikova, Sergejs Nikisins, Janine Reiche, Wei Cai, Adam Meijer, Anne Teirlinck, Amparo Larrauri, Inmaculada Casas, Vincent Enouf, Sophie Vaux, Frederikke Kristensen Lomholt, Ramona Trebbien, Helena Jirincova, Helena Sebestova, Mónika Rózsa, Zsuzsanna Molnár, Gudrun Aspelund, Gudrun Erna Baldvinsdottir, Simon Cottrell, Catherine Moore, Athanasios Kossyvakis, Kassiani Mellou, Olga Sadikova, Johanna Kristina Tamm, Nathalie Bossuyt, Isabelle Thomas, Edita Staroňová, Lyudmila Kudasheva, Boris Pleshkov, Niina Ikonen, Otto Helve, Emma Dickson, Tanya Curran, Kseniya Komissarova, Kirill Stolyarov, Veronika Vysotskaya, Natallia Shmialiova, Božidarka Rakočević, Danijela Vujošević, Romella Abovyan, Shushan Sargsyan, Khatuna Zakhashvili, Ann Machablishvili, Oksana Koshalko, Iryna Demchyshyna, Michal Mandelboim, Aharona Glatman‐Freedman, Rory Gunson, Shivani Karanwal, Raquel Guiomar, Ana Paula Rodrigues, Charlene Bennett, Lisa Domegan, Arijana Kalaveshi, Xhevat Jakupi, Gurbangul Ovliyakulova, Neli Korsun, Nadezhda Vladimirova

**Affiliations:** ^1^ World Health Organization (WHO) Regional Office for Europe Copenhagen Denmark; ^2^ UK Health Security Agency London UK; ^3^ National Institute of Public Health Bucharest Romania; ^4^ Cantacuzino National Military‐Medical Institute for Research and Development Bucharest Romania; ^5^ Clinical Center University of Sarajevo Sarajevo Bosnia and Herzegovina; ^6^ Public Health Institute of the Republic of Srpska Banja Luka Bosnia and Herzegovina; ^7^ Faculty of Medicine University of Banja Luka Banja Luka Bosnia and Herzegovina; ^8^ General Directorate of Public Health Ankara Türkiye; ^9^ National Research Institute Warsaw Poland; ^10^ National Public Health Center under the Ministry of Health Vilnius Lithuania; ^11^ National Public Health Surveillance Laboratory Vilnius Lithuania; ^12^ Ministry for Health Regulation Pieta Malta; ^13^ University of Geneva Hospitals Geneva Switzerland; ^14^ Laboratoire National de Santé Dudelange Luxembourg; ^15^ Croatian Institute of Public Health Zagreb Croatia; ^16^ National Laboratory of Health, Environment and Food Ljubljana Slovenia; ^17^ National Institute of Public Health Ljubljana Slovenia; ^18^ Institute of Virology, Vaccines and Sera Torlak Belgrade Serbia; ^19^ Institute of Public Health of Serbia Belgrade Serbia; ^20^ National Agency for Public Health Chişinău Republic of Moldova; ^21^ Centre for Disease Prevention and Control Riga Latvia; ^22^ Riga East University Hospital Riga Latvia; ^23^ Robert Koch Institute Berlin Germany; ^24^ Centre for Infectious Disease Control Bilthoven The Netherlands; ^25^ Carlos III Health Institute Madrid Spain; ^26^ Institut Pasteur Paris France; ^27^ Santé Publique France Saint‐Maurice France; ^28^ Statens Serum Institute Copenhagen Denmark; ^29^ National Institute of Public Health Prague Czechia; ^30^ National Public Health Center Budapest Hungary; ^31^ The Directorate of Health Reykjavik Iceland; ^32^ The National University Hospital of Iceland Reykjavik Iceland; ^33^ Public Health Wales Cardiff UK; ^34^ Hellenic Pasteur Institute Athens Greece; ^35^ National Public Health Organization Athens Greece; ^36^ The Estonian Health Board Tallinn Estonia; ^37^ Sciensano Brussels Belgium; ^38^ Public Health Authority of the Slovak Republic Bratislava Slovakia; ^39^ Sanitary and Epidemiological Welfare and Public Health Service of the Republic of Uzbekistan Toshkent Uzbekistan; ^40^ Finnish Institute for Health and Welfare Helsinki Finland; ^41^ Public Health Agency Belfast UK; ^42^ Belfast Health and Social Care Trust Belfast UK; ^43^ Smorodintsev Research Institute of Influenza St Petersburg Russian Federation; ^44^ Republican Center for Hygiene, Epidemiology and Public Health Minsk Belarus; ^45^ Republican Research and Practical Center for Epidemiology and Microbiology Minsk Belarus; ^46^ Institute of Public Health of Montenegro Podgorica Montenegro; ^47^ National Center for Disease Control and Prevention Yerevan Armenia; ^48^ National Center for Disease Control and Public Health Tbilisi Georgia; ^49^ Public Health Center of the Ministry of Health of Ukraine Kyiv Ukraine; ^50^ Israel Ministry of Health Ramat Gan Israel; ^51^ West of Scotland Specialist Virology Centre Glasgow UK; ^52^ Public Health Scotland Glasgow UK; ^53^ National Institute of Health Doctor Ricardo Jorge Lisbon Portugal; ^54^ National Virus Reference Laboratory Dublin Ireland; ^55^ Health Protection Surveillance Centre Dublin Ireland; ^56^ National Institute of Public Health of Kosovo Prishtina Kosovo; ^57^ Ministry of Health and Medical Industry of Turkmenistan Ashgabat Turkmenistan; ^58^ National Center of Infectious and Parasitic Diseases Sofia Bulgaria

**Keywords:** COVID‐19 pandemic, epidemiology, Europe, respiratory syncytial virus, severity, surveillance

## Abstract

**Background:**

The emergence of the Severe Acute Respiratory Syndrome Coronavirus 2 (SARS‐CoV‐2) in early 2020 and subsequent implementation of public health and social measures (PHSM) disrupted the epidemiology of respiratory viruses. This work describes the epidemiology of respiratory syncytial virus (RSV) observed during two winter seasons (weeks 40–20) and inter‐seasonal periods (weeks 21–39) during the pandemic between October 2020 and September 2022.

**Methods:**

Using data submitted to The European Surveillance System (TESSy) by countries or territories in the World Health Organization (WHO) European Region between weeks 40/2020 and 39/2022, we aggregated country‐specific weekly RSV counts of sentinel, non‐sentinel and Severe Acute Respiratory Infection (SARI) surveillance specimens and calculated percentage positivity. Results for both 2020/21 and 2021/22 seasons and inter‐seasons were compared with pre‐pandemic 2016/17 to 2019/20 seasons and inter‐seasons.

**Results:**

Although more specimens were tested than in pre‐COVID‐19 pandemic seasons, very few RSV detections were reported during the 2020/21 season in all surveillance systems. During the 2021 inter‐season, a gradual increase in detections was observed in all systems. In 2021/22, all systems saw early peaks of RSV infection, and during the 2022 inter‐seasonal period, patterns of detections were closer to those seen before the COVID‐19 pandemic.

**Conclusion:**

RSV surveillance continued throughout the COVID‐19 pandemic, with an initial reduction in transmission, followed by very high and out‐of‐season RSV circulation (summer 2021) and then an early start of the 2021/22 season. As of the 2022/23 season, RSV circulation had not yet normalised.

## 1 INTRODUCTION

In temperate regions, respiratory syncytial virus (RSV) typically circulates in the winter months, causing more severe illness particularly in infants and older adults that often results in hospitalisation, including admission to intensive care units.^1^, ^2^ There are often periods of co‐circulation with influenza and other seasonal respiratory viruses. RSV is monitored through sentinel and/or non‐sentinel surveillance in many countries, territories and areas (henceforth referred to as countries) in the World Health Organization (WHO) European Region, the European Union (EU) and European Economic Area (EEA) countries (hereafter referred to as Europe). These sentinel surveillance systems were originally established for influenza and previously described.^3^ A new vaccine has been approved in September 2023 by the European Medicines Agency for use in older adults and infants under 6 months of age. Monoclonal immunoglobulins are available as prophylaxis during the typical period of RSV circulation to protect young infants during their first RSV season, especially those with chronic underlying heart and lung disease at higher risk of severe disease.^4^

The emergence and spread of Severe Acute Respiratory Syndrome Coronavirus 2 (SARS‐CoV‐2) in early 2020 and the subsequent public health and social measures (PHSM) implemented by Member states to reduce its transmission and anticipated morbidity and mortality have disrupted the spread of other respiratory viruses, including RSV, in both the southern^5^, ^6^, ^7^ and northern hemispheres.^8^, ^9^, ^10^

This work aims to describe the epidemiology of RSV in Europe during the 2020/21 and 2021/22 winter seasons [weeks 40/2020 (late September) to 20/2021 (mid‐May) and 40/2021 to 20/2022] and two inter‐seasonal periods (weeks 21 to 39/2021 and 21 to 39/2022) through three surveillance systems [primary care sentinel, secondary care Severe Acute Respiratory Infection (SARI) and non‐sentinel surveillance] in comparison with historical pre‐COVID‐19 pandemic data.

## 2 METHODS

This retrospective epidemiological analysis of RSV used weekly data submitted to The European Surveillance System (TESSy) by the European Regional surveillance network jointly coordinated by ECDC and the WHO Regional Office for Europe. As RSV is not notifiable, reporting is voluntary. Weekly counts of detections and specimens tested in 48 reporting countries between week 40/2020 and week 39/2022 were downloaded on 14 October 2022. Countries were included in this analysis if at least one sample tested was reported.

Using specimens taken from selected cases of influenza‐like illness (ILI) and acute respiratory infection (ARI) presenting to primary care sentinel surveillance system (these systems have been described previously^3^), we calculated the aggregated weekly counts of RSV detections and tests and the percentage positivity when at least 10 tests were performed. These counts and percentages were compared with the four previous seasons (2016/17 to 2019/20; hereafter referred to as pre‐COVID‐19 pandemic seasons). Using weekly RSV percentage positivity from four pre‐COVID‐19 pandemic seasons of sentinel data, we used the Moving Epidemic Method (MEM) as described to calculate RSV‐specific epidemic thresholds for each country (Table S2) and identify potential changes in seasonal patterns, as previously shown.^11^ It is important to note that these thresholds may differ from those used in the country for surveillance purposes. Epidemic activity was determined to have started when percentage positivity was above the country‐specific epidemic threshold for the first of at least two consecutive weeks. The duration of the epidemic period was defined as the total number of weeks above the threshold (including non‐consecutive weeks).

Similarly, aggregated weekly counts of detections and specimens tested were calculated, along with percentage positivity, for SARI sentinel surveillance (hospital inpatients meeting the case definition). Only two countries reported data in 2020/21 and 16 in 2021/22 periods; historical data regarding number of tests performed were unavailable for this surveillance system. Data included aggregated SARI cases by age group (≤4 years, 5–14 years, 15–64 years and ≥65 years).

For the non‐sentinel surveillance system, only the weekly counts of detections were aggregated but percentage positivity could not always be calculated given the unavailability and/or uncertainty around some of the denominators. Specimens from this system were taken from patients that originated from hospitals, schools, primary care facilities not involved in sentinel surveillance, or nursing homes and other institutions.

For all three systems, data in the study period were compared with historical data. Some countries stopped surveillance or reporting data to TESSy over the summer months for some surveillance systems considered. All analyses were conducted in R version 4.0.5.^12^

## 3 RESULTS

### 3.1 Primary care sentinel surveillance

**FIGURE 1:**
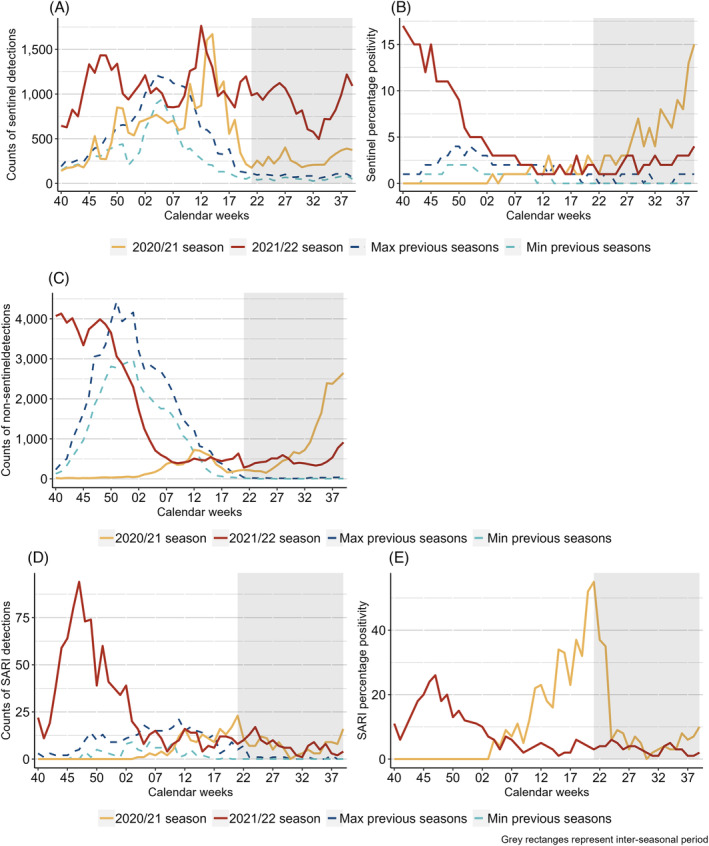
Top row: Count of specimens tested (A) and percentage positivity (B) of RSV specimens per week from sentinel sources compared with the minimum (Min) and maximum (Max) from pre‐COVID‐19 pandemic seasons; middle row: count of non‐sentinel RSV detections compared to the minimum (Min) and maximum (Max) from pre‐COVID‐19 pandemic seasons (C); bottom row: count of specimens tested for RSV (D) and percentage positivity (E) from SARI sites across the selection of countries included, over several seasons. Note: percentage positivity was calculated when at least 10 specimens were tested; grey rectangles represent inter‐seasonal periods; seasons represent the time period ranging from week 40 to 39.

When compared with pre‐COVID‐19 pandemic seasons, there was an increase in the number of specimens tested for RSV in both the 2020/21 and the 2021/22 seasons: 21,803 and 36,040, respectively, compared with an annual average of 15,796 in pre‐COVID‐19 pandemic seasons (from 21 countries on average), representing a 38% and 128% increase, respectively. More countries reported during the 2021/22 season than during the 2020/21 and pre‐COVID‐19 pandemic seasons (Figure 1A,B and Table 1).

**TABLE 1:** Summary breakdown of RSV data by surveillance system for the 2020/21 (weeks 40/2020 to 39/2021) and 2021/22 (weeks 40/2021 to 39/2022) seasons and corresponding inter‐seasonal periods, with percentage positivity in brackets, and previous seasons (seasonal and inter‐seasonal periods), in the selection of countries included.

		2021/22 season		2020/21 season		2016/17–2019/20 seasons			
		Seasonal period	Inter‐seasonal period	Seasonal period	Inter‐seasonal period	Mean during seasonal period	Range during seasonal period	Mean during inter‐seasonal period	Range during inter‐seasonal period
Sentinel surveillance									
Number of countries^a^		34	27	19	17	‐	18–23	‐	7–12
Specimens tested	Regional totals	36,040	19,197	21,803	5076	15,796	13,853–18,026	1338	1121–1532
	Country ranges	0–5688	3–8031	1–4723	1–2477	2–4502	2–5654	2–542	1–731
Detections	Regional totals	2018 (6%)	375 (2%)	195 (1%)	316 (6%)	1669 (11%)	1461–1905	15 (1%)	4–26
	Country ranges	0–635 (0%–15%)	0–103 (0%–15%)	0–78 (0%–7%)	0–175 (0%–22%)	0–312 (0%–50%)^b^	0–430	0–11 (0%–2%)	0–20
Non‐sentinel surveillance									
Number of countries^a^		34	24	18	20	‐	30–33	‐	9–15
Detections	Regional totals	62,426	8876	6966	18,325	49,738	44,081–55,614	220	118–426
	Country ranges	0–14,071	0–1955	0–5454	0–6983	0–11,888	0–16,456	0–61	0–164
SARI surveillance									
Number of countries^a^		16	11	2	7	‐	5–10	‐	1–3
Specimens tested	Regional totals	9478	4238	682	1983	‐	‐	‐	‐
	Country ranges	28–2370	2–1630	94–588	27–736	‐	‐	‐	‐
Detections	Regional totals	948 (10%)	136 (3%)	144 (21%)	148 (7%)	200	178–227	7	3–15
	Country ranges	0–355 (0%–60%)	0–98 (0%–18%)	0–144 (0%–24%)	0–55 (0%–25%)	0–93	0–105	0–3	0–9

^a^ Reporting at least one specimen tested.

^b^ Note that the 50% was based on 10 specimens tested in one country.

A total of 19 countries reported only 195 RSV detections from a total of 21,803 primary care sentinel surveillance specimens tested during the 2020/21 seasonal period. This represents an overall positivity of 1%, ranging up to 7% in France, which was lower than in the four pre‐COVID‐19 pandemic seasons (mean of 1669 positive specimens, 11% positivity from an average of 21 countries) (Figure 1A,B and Table 1). Only seven countries reported at least five detections during the 2020/21 season (Table S1). Only France and Switzerland saw clear waves of activity. Between weeks 5/2020 and 19/2021 in France, percentage positivity was above 10% for all but 2 weeks, whereas Switzerland saw percentage positivity between 4% and 17% between weeks 16 and 20/2021 (Table S1).

**FIGURE 2:**
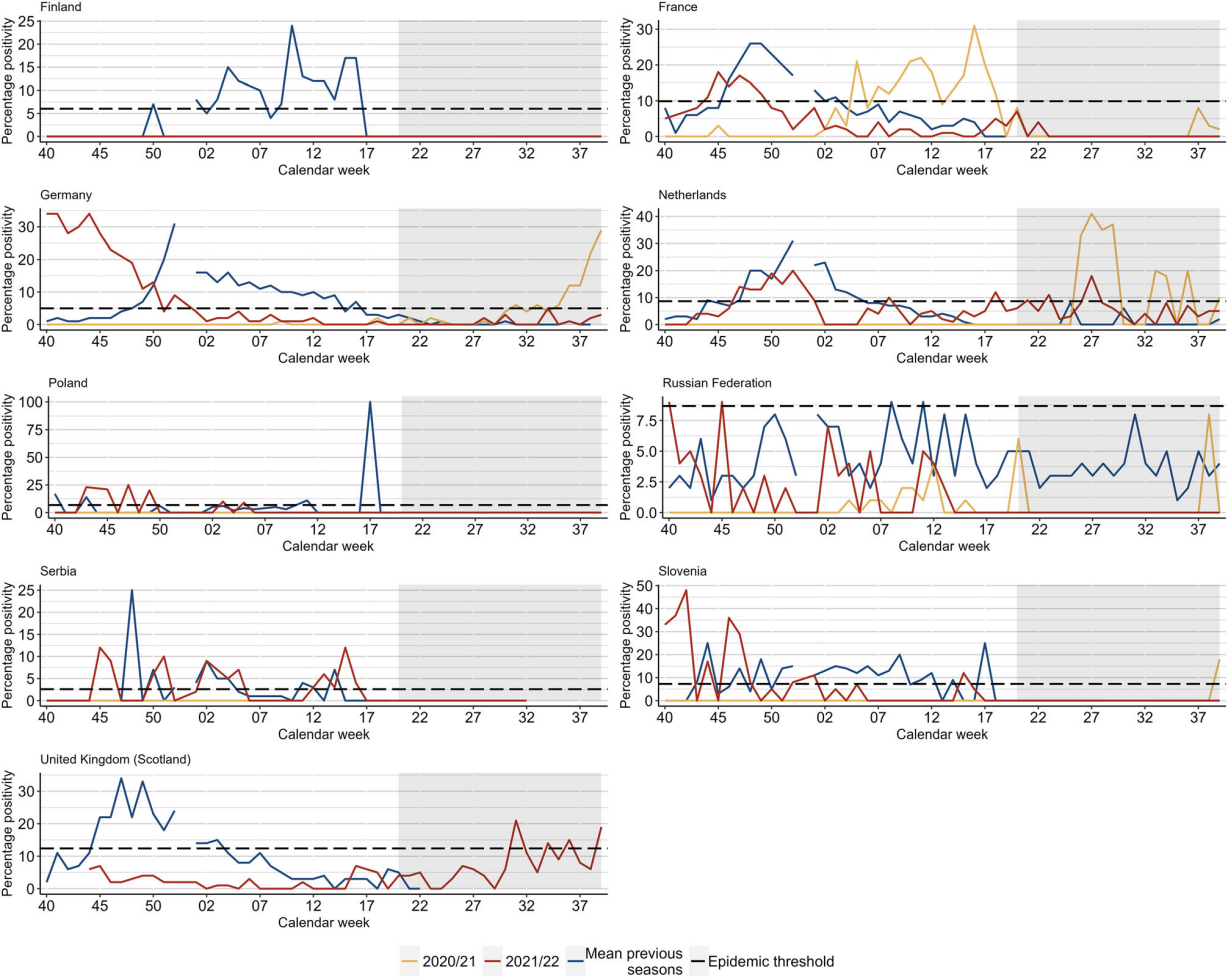
Summary of Moving Epidemic Method (MEM) calculations (calculated by authors) comparing percentage positivity in pre‐COVID‐19 pandemic seasons (2016/17 to 2019/20) and 2020/21 (weeks 40/2020 to 39/2021) and 2021/22 (weeks 40/2021 to 39/2022) seasons, where possible in the selection of countries included. Horizontal lines represent the epidemic threshold for each season where it could be calculated. Note: grey rectangles represent inter‐seasonal periods; the same epidemic threshold calculations were used for the 2021/22 and 2020/21 season. More detailed results can be found in Table S2.

During the 2020/21 season, of the nine countries where the epidemic thresholds could be calculated, only France reported peaks of RSV positivity above its threshold. France, Germany and Slovenia experienced early starts to their 2021/22 seasonal period compared with the average starting week of pre‐COVID‐19 pandemic seasons (Figure 2).

During the 2021 inter‐seasonal period, a total of 316 positive specimens from 5076 specimens tested (6% positivity) were reported from 17 countries (Figure 1A,B and Table 1). A total of nine countries [Estonia (*n* = 8, 5% positivity), France (*n* = 5, 4%), Georgia (*n* = 21, 3%), Germany (*n* = 175, 7%), Ireland (*n* = 7, 2%), the Netherlands (*n* = 39, 22%), Slovenia (*n* = 16, 16%), Switzerland (*n* = 35, 9%) and Ukraine (*n* = 5, 4%)] saw higher than usual activity (Figure S1). Of the countries with inter‐seasonal activity, three (Germany, the Netherlands and Slovenia) exceeded their MEM epidemic threshold, with only the Netherlands having experienced activity during this inter‐seasonal time period in pre‐COVID‐19 pandemic seasons. This represents only sporadic detections, as numbers of collected samples are generally low in the inter‐seasonal time period (Figure 2 and Figure S1).

During the 2021/22 season, 34 countries reported 2018 detections from 36,040 tests performed (6% positivity). This was higher than during the 2020/21 season, but lower than during the pre‐COVID‐19 pandemic seasons (Figure 1A,B and Table 1). A peak of 17% positivity was observed in week 40/2021 which then fell to 3% (week 2/2022) and remained below this level until the end of the season. Regionally, this peak was higher than in the pre‐COVID‐19 pandemic season when regional positivity did not rise above 5% for any given week (Figure 1B and Table 1). In six countries [Denmark, Slovenia, Sweden, United Kingdom (Northern Ireland, Scotland and Wales)], the start of the 2021/22 season was already discernible in the latter part of the 2021 inter‐seasonal period with detections peaking on or around week 40/2021. Where possible to compare with pre‐COVID‐19 pandemic seasons (*n* = 23 countries), 14 countries experienced similar or higher positivity. Positive specimens continued to be detected in several countries during the second half of the season leading into the 2022 inter‐seasonal period, most notably in Georgia, the Netherlands, Spain and Switzerland (Figure S1B).

During the 2022 inter‐seasonal period, 375 detections were reported from 19,197 samples tested (2% positivity) from 27 countries. Both the number of tests performed and positivity for this period were higher than seen in pre‐COVID‐19 pandemic inter‐seasons: up to 1336 tests and up to 1% positivity (Table 1). Eight countries reported RSV activity outside of the characteristic winter season with at least five detections: Denmark (*n* = 59, 3% positivity), Georgia (*n* = 33, 5%), Germany (*n* = 15, 1%), the Netherlands (*n* = 40, 6%), Spain (*n* = 103, 1%), Switzerland (*n* = 9, 1%), United Kingdom (Scotland) (*n* = 86, 9%) and Slovenia (*n* = 12, 15%) (Figure S1 and Table S1). During this period, only the Netherlands and the United Kingdom (Scotland) experienced RSV positivity reaching the epidemic levels previously calculated (Figure 2).

### 3.2 Non‐sentinel surveillance

A total of 18 countries reported at least one non‐sentinel RSV detection in the 2020/21 season, with a total of 6966 detections. This compares with an average of 49,738 detections from 32 countries in pre‐COVID‐19 pandemic seasons (Figure 1C and Table 1). Between weeks 40/2020 and 1/2021, fewer than 60 weekly detections were recorded, much less than the weekly average during the pre‐COVID‐19 pandemic seasons for the same weeks: 180 (week 40) to 3437 (week 1). Detections peaked in week 12/2021 (Iceland and Ukraine) with 722 RSV detections and fell thereafter until week 18/2021. Between weeks 12 and 18/2021, the number of detections was comparable with pre‐COVID‐19 pandemic seasons (Figure 1C). For the countries with at least one historical season for comparison (*n* = 17), only Iceland and Ukraine experienced more detections (Table S2) and Finland, Greece, Hungary, Poland, Portugal, Republic of Moldova, Serbia and the United Kingdom saw an earlier peak in 2020/21 than in pre‐COVID‐19 pandemic seasons.

The 2021 inter‐season saw 18,325 RSV detections reported from 20 countries, all of which could be compared with pre‐COVID‐19 pandemic seasons and mostly had higher numbers in 2021 in comparable weeks (average of 220 detections from 13 countries), with the exceptions of Estonia and Latvia (Figure 1C and Tables 1 and S2). This period was characterised by a gradual rise in detections (peaking in week 39/2021 with 2646 detections), which had previously not been reported.

During the 2021/22 season, 62,426 detections were reported from 34 countries, of which 32 could be compared with pre‐COVID‐19 pandemic seasons (Figure 1 and Table 1). The start of the 2021/22 season was a continuation of the high levels of detections already seen during the 2021 inter‐seasonal period, with two peaks of detections: weeks 40/2021 [observed in Denmark, Slovenia, United Kingdom (Northern Ireland, Scotland and Wales)] and 46/2021 (observed in France and Spain). From week 48/2021 until week 7/2022, the numbers of positive detections then gradually fell to levels lower than in pre‐COVID‐19 pandemic seasons. Between weeks 17 and 20/2022, the weekly average of detections was 514, higher than any pre‐COVID‐19 pandemic season (Figure 1C). Of the countries with historical comparisons, nine recorded fewer, 10 recorded more and 13 recorded a similar number of detections than in pre‐COVID‐19 pandemic seasons (Table S2).

Most countries recorded unusually high and/or early peaks of activity during the 2021/22 season. Some countries (Iceland and the Netherlands) saw a biphasic seasonal activity. Others [Denmark, Portugal, Russian Federation, Slovenia, United Kingdom (England, Scotland and Wales)] saw their number of detections decline after an early peak but then rise into the 2022 inter‐seasonal period (Table S2).

The 2022 inter‐season saw 8876 RSV detections from 24 countries, of which 18 had historical data for comparison (Figure 1C and Tables 1 and Table S2). Bulgaria, Iceland, Portugal, Russian Federation and the United Kingdom (Northern Ireland and Scotland) observed more detections than during pre‐COVID‐19 pandemic inter‐seasons but without a distinct peak of activity (Figure S2). In contrast, the Netherlands and the United Kingdom (Wales) did experience a peak of detections in weeks 23/2022 and 28/2022, respectively. Denmark, France, Slovenia and Spain reported a rise in activity late in the period and leading into the 2022/23 season (Figure S2).

### 3.3 SARI surveillance

During the 2020/21 season, only Belgium and Malta reported SARI data, but only Belgium reported 144 cases identified among 682 patients tested (21% positivity). This compares with an average of 200 detections from seven countries in pre‐COVID‐19 pandemic seasons (Table 1). Detections peaked in week 20/2021 with 55% positivity (*n* = 17) (Figure 1D,E). Of the 480 patients with known age (all from Belgium), 136 tested positive (28% positivity), with children ≤4 years representing 90% of patients (41% positivity). Children aged 5–14 years saw 26% positivity, which was higher than in the oldest age group (7%) (Table 2).

**TABLE 2:** Number of SARI detections (and percentage positivity when at least 10 specimens were tested per age group) per country by age group (in years).

Country	2022 inter‐season					2021/22 season					2021 inter‐season					2020/21 season				
	0–4	5–14	15–64	≥65	Total	0–4	5–14	15–64	≥65	Total	0–4	5–14	15–64	≥65	Total	0–4	5–14	15–64	≥65	Total
Armenia	2 (5%)	0 (0%)	0 (0%)	0 (−%)	2 (2%)	9 (6%)	2 (3%)	0 (0%)	0 (−%)	11 (3%)	11 (12%)	0 (0%)	0 (0%)	0 (−%)	11 (5%)					
Belarus	0 (0%)	0 (0%)	0 (−%)	0 (−%)	0 (0%)	5 (9%)	1 (3%)	0 (−%)	0 (−%)	6 (7%)										
Belgium^a^	14 (35%)	0 (−%)	0 (−%)	0 (0%)	14 (16%)	70 (20%)	4 (8%)	5 (2%)	4 (1%)	83 (9%)	41 (39%)	5 (45%)	0 (0%)	7 (14%)	53 (28%)	122 (41%)	5 (26%)	0 (0%)	9 (7%)	136 (28%)
Bosnia and Herzegovina						6 (24%)	0 (0%)	0 (−%)	0 (−%)	6 (17%)										
Croatia^a^	4 (7%)	0 (0%)	0 (−%)	0 (0%)	4 (1%)	6 (9%)	0 (0%)	1 (−%)	1 (0%)	8 (2%)										
Malta	0 (−%)	0 (−%)	0 (−%)	1 (1%)	1 (1%)	0 (−%)	0 (−%)	0 (−%)	8 (3%)	8 (3%)	0 (−%)	0 (−%)	0 (−%)	1 (1%)	1 (1%)	0 (−%)	0 (−%)	0 (−%)	1 (0%)	0 (0%)
Montenegro						24 (67%)	2 (−%)	1 (−%)	0 (−%)	27 (60%)										
Republic of Moldova	0 (−%)	0 (−%)	0 (−%)	0 (−%)	0 (−%)	21 (26%)	2 (8%)	4 (6%)	0 (0%)	27 (12%)										
Russian Federation	5 (2%)	1 (1%)	0 (−%)	0 (−%)	6 (2%)	40 (9%)	6 (3%)	1 (1%)	0 (−%)	47 (6%)	18 (11%)	2 (5%)	1 (2%)	0 (−%)	21 (8%)					
Türkiye	4 (1%)	0 (0%)	1 (1%)	0 (−%)	5 (1%)	258 (22%)	8 (4%)	8 (3%)	0 (−%)	274 (17%)										
Ukraine						3 (17%)	2 (−%)	0 (0%)	0 (−%)	5 (7%)	2 (4%)	1 (8%)	0 (0%)	0 (−%)	3 (2%)					
Uzbekistan	0 (0%)	0 (0%)	0 (−%)	0 (−%)	0 (0%)	64 (29%)	1 (3%)	2 (73%)	0 (−%)	67 (24%)										
**Total**	**29 (4%)**	**1 (0%)**	**1 (1%)**	**1 (0%)**	**32 (2%)**	**506 (20%)**	**28 (4%)**	**22 (2%)**	**13 (1%)**	**569 (11%)**	**72 (18%)**	**8 (10%)**	**1 (0%)**	**8 (6%)**	**89 (10%)**	**122 (41%)**	**5 (26%)**	**0 (0%)**	**9 (5%)**	**136 (25%)**

^a^ Country data have been updated upon request after data collection date.

**FIGURE 3:**
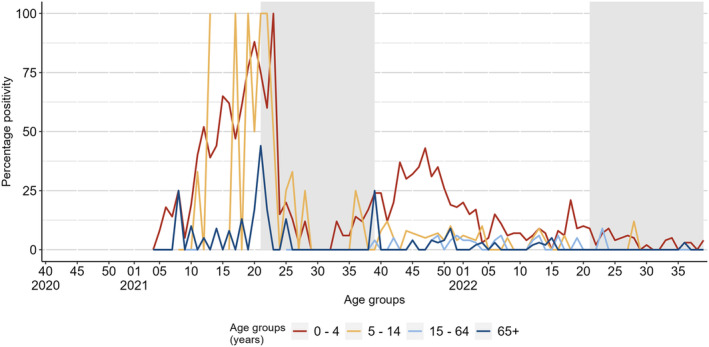
Percentage positivity of positive specimens detected from SARI sites per age group, from the selection of countries included. Grey rectangles represent inter‐seasonal periods.

During the 2021 inter‐season, seven countries (Armenia, Belgium, Georgia, Ireland, Malta, Russian Federation and Ukraine) reported SARI data with a total of 148 RSV detections from 1983 patients (7% positivity). This compares with up to 15 detections from up to three countries in pre‐COVID‐19 pandemic seasons (Tables 1 and S1). Between weeks 24 and 37/2021, the percentage positivity ranged between 2% and 9%, except for week 30/2021 when no detections were reported. Percentage positivity peaked in week 39/2021 at 10%, leading into the 2021/22 season (Figures 1E and S3). When possible to compare with sentinel systems (Georgia, Ireland, Russian Federation and Ukraine), all countries but Ireland saw a delayed peak in SARI positivity after the one in sentinel detections. Among 898 patients with known age tested in five countries, 89 (10%) were positive. Children ≤4 years accounted for 81% (*n* = 72) of all cases with known age, and 18% of their samples tested positive for RSV. Only Belgium reported over 10% positivity in another age group (those ≥65 years) (Figure 3 and Table 2).

During the 2021/22 season, 948 SARI cases were reported among 9478 patients tested (10% positivity) in 16 countries (Figure 1D,E and Table 1). A peak of 25% positivity was observed in week 47/2021 in Georgia and Türkiye, after which it fell until week 3/2022 and mostly ranged between 1% and 5% until the end of the season (Figure 1E). Among 5220 patients with known age, 569 detections (11%) were reported by 12 countries. These included 506 (89%) in children ≤4 years and 13 (2%) in persons ≥65 years with a percentage positivity of 20% and 1%, respectively (Table 2). Positive specimens in the youngest age group peaked at the start of the season and in week 18/2022 (Figure 3).

During the 2022 inter‐season, 136 RSV detections were reported from 4238 patients tested (3% positivity) in 11 countries, with positivity ranging between 1% and 6% and peaking in week 24/2022 (Table 1, Figure 1E, Table S1 and Figure S3). Of the 32 cases reported with known age, 29 were ≤4 years from six countries (4% positivity) (Table 2).

## 4 DISCUSSION

We showed that the COVID‐19 pandemic and its response have impacted the circulation of RSV in Europe since the autumn of 2020. Very little circulation was seen during the 2020/21 season, but unusual inter‐seasonal activity was seen in the summer of 2021, followed by early and high peaks of activity during the 2021/22 season as observed in all surveillance systems. This atypical inter‐seasonal activity continued in many countries in the summer months of 2022. Hospitalised cases of RSV have been predominantly in those aged ≤4 years.

Although countries included in this analysis have tested more specimens from sentinel surveillance systems for RSV during the COVID‐19 pandemic than in prior seasons, fewer viruses were detected during the usual weeks of RSV circulation of the 2020/21 season. The similar findings from three separate surveillance systems (primary care sentinel, non‐sentinel and SARI) and a higher number of countries reporting compared with pre‐COVID‐19 pandemic seasons provide some assurance that this is a true observation of reduced circulation of RSV, likely due to the impact of public health and social measures in the early phase of the pandemic rather than a change in testing practices.

Many countries also observed an unusual increase in RSV activity in the summer months of 2021 and 2022, representing out‐of‐season activity, in all systems, usually translating into an early start of the next epidemic season. In primary care sites in some countries (Denmark, Germany and Slovenia, among others), the 2021/22 season began earlier than during pre‐COVID‐19‐pandemic seasons. In other parts of the Region (such as in France, Ireland and the Netherlands, among others), seasonality returned to similar timing to those observed in pre‐COVID‐19‐pandemic seasons, but with much higher positivity. Both observations may be associated with the lack of RSV circulation observed during the 2020/21 season and hence a widespread lack of exposure to RSV, particularly in younger cohorts, resulting in the build‐up of an increased pool of susceptibles.

When comparing SARI activity to historical data, a higher peak of detections was seen late in the 2020/21 season and early during the 2021/22 season. Because of a lack of historical denominator data, it was not possible to compare the patterns of positivity. Children aged ≤4 years were disproportionately more affected than any other age group, accounting for at least 89% of all SARI cases during all four periods considered whereas those aged ≥65 years accounted for 2% of cases, fewer than would be expected.^13^ This may be a result of PHSM against COVID‐19 and continued behavioural changes, especially in older generations, such as continued social distancing or mask wearing even after measures have been lifted. However, it may also be explained by some countries (in Eastern Europe and Central Asia) having a lower threshold for hospitalising young children than other age groups^3^ or changing their testing practices during the COVID‐19 pandemic.

Previous studies have looked at the importance of RSV infection causing hospitalisation in the first 2 years of life.^4^, ^14^ Given that children born during the COVID‐19 pandemic are unlikely to have been exposed to RSV in their first or second year of life, it is possible that when they are older, the severity of a primary RSV infection is reduced. However, it is also possible that the number of children hospitalised during the 2022/23 season will still be greater because of the large number of RSV‐naïve children born since the start of the pandemic, resulting in a high burden of disease and hospitalisation rate.^6^ Combined with hospitalisations resulting from other respiratory viruses across all age groups, RSV has the potential to cause high pressure on healthcare systems across the Region.^5^

This significant shift in circulation patterns was also observed in other parts of the world where RSV typically also circulates during the winter months such as in Australia and New Zealand^6^, ^8^ or South Africa.^5^, ^6^, ^8^, ^15^ RSV circulation was not uniform across these countries, with some experiencing stronger than expected out‐of‐season activity,^5^ whereas others experienced delayed or continued circulation.^6^ A delayed circulation of RSV during the summer months had previously been reported after the 2009 A(H1N1) influenza pandemic.^16^ The overall reduction in transmission was a very possible result of PHSM implemented (such as mask wearing, stay‐at‐home orders and school orders) to restrict the spread of SARS‐CoV‐2 within and between countries^7^ by breaking transmission chains. We know that this implementation was neither uniform between countries nor over time and may be an important factor; similar results were found by other groups.^17^

This analysis contains several limitations, including the limited and varying number of countries that report data through various surveillance systems, some of which are not an integral part of routine surveillance but have been part of ECDC projects initiated during the COVID‐19 pandemic. Changes in testing practices and number of reporting sites because of the COVID‐19 pandemic were not considered here. The MEM thresholds were based on only four historical seasons, and very few countries could be included because of the intermittent absence of weekly data or denominators. The interpretation of the calculated epidemic thresholds should therefore be taken with caution and does not necessarily represent the national epidemic thresholds that are set by the individual countries. There were too few countries for a robust investigation into geo‐temporal patterns of increased activity across countries in the Region. The lack of non‐sentinel denominator data did not allow systematic percentage positivity calculations, a potential weakness of this data source when interpreted alone. However, the consistency of non‐sentinel trends with those from other surveillance systems provides some reassurance to the validity of the results. Additionally, the availability of age data for sentinel, non‐sentinel and historical SARI surveillance systems would have allowed a better understanding of which age groups have been most affected by these unusual circulation patterns and whether children were overrepresented. Finally, the limited number of countries reporting SARI age data may not be representative of the Region (also given that these countries are mostly located in its Eastern part) or of the age distribution of patients at large (it is unclear if cases have an a priori equal chance of being tested for RSV regardless of their age). Unfortunately, the absence of historical SARI denominator data and the large age groups into which cases are grouped do not allow for a more detailed interpretation of our results. In addition, during the COVID‐19 pandemic, there were varying degrees of disruption to national sentinel surveillance systems because of changes in health‐seeking behaviours and limitations in the capacity of sites to receive cases and take specimens, impacting the ability of these systems to monitor respiratory viruses, including RSV.

Despite these limitations, this descriptive study illustrates how the emergence of a novel respiratory virus and associated public health measures can significantly disrupt the circulation of established seasonal respiratory viruses. It is likely that the absence of circulation of seasonal respiratory pathogens during a season led to out‐of‐season activity and, potentially, to larger‐than‐expected peaks of activity thereafter, as observed in summer 2021 and the early 2021/22 season. RSV activity during the 2022/23 season continues to follow unusual patterns, and the implementation of integrated surveillance systems monitoring multiple respiratory viruses simultaneously may prove invaluable to both monitor RSV and other seasonal viral respiratory infections and also in planning for the future emergence of a novel pandemic respiratory virus.

## 5 CONCLUSION

The emergence and spread of SARS‐CoV‐2 and associated control measures in the EU/EEA and in the WHO European Region have significantly impacted the spread and timing of seasonal respiratory viruses such as RSV. For the last two winter seasons, and for the summer periods in between, much higher proportions of RSV detections or a temporal shift in transmissions were detected compared with pre‐COVID‐19 pandemic seasons. Further work is required to determine the possible explanatory factors including the implementation and relaxation in PHSM and factors such as possible SARS‐CoV‐2 viral interference to this shift in timing, activity, and potential difference in age distribution of RSV cases. These results highlight the importance of being aware of and preparing for continued potential unusual patterns in the epidemiology of RSV (and other respiratory viruses). It is also a lesson learned with regard to the impact of future emergence and spread of respiratory viruses with pandemic potential on the epidemiology of seasonal respiratory viruses.

## AUTHOR CONTRIBUTIONS

**Margaux Marie Isabelle Mesle:** Conceptualization; formal analysis; investigation; methodology; validation; visualization; writing—original draft; writing—review and editing. **Mary Sinnathamby:** Formal analysis; investigation; methodology; writing—original draft; writing—review and editing. **Piers Mook:** Conceptualization; formal analysis; funding acquisition; investigation; methodology; project administration; supervision; validation; visualization; writing—review and editing. **WHO European Region respiratory network WHO European Region respiratory network:** Writing—review and editing. **Richard Pebody:** Conceptualization; funding acquisition; methodology; project administration; supervision; validation; writing—review and editing.

## CONFLICT OF INTEREST STATEMENT

The following authors declare having received funding from the Innovative Medicines Initiative (IMI): Adam Meijer (The Netherlands) and Anne Teirlinck (The Netherlands). All other authors have no conflicts of interest to declare.

### PEER REVIEW

The peer review history for this article is available at https://www.webofscience.com/api/gateway/wos/peer-review/10.1111/irv.13219.

## DISCLAIMER

The authors affiliated with the World Health Organization (WHO) are solely responsible for the views expressed in this publication and do not necessarily represent the decisions or policies of the WHO.

## Supporting information


**Figure S1:** Counts (A) and percentage positivity (B) of RSV‐positive specimens detected from sentinel sources by country and season, from the selection of countries included. Previous seasons range from 2016/17 to 2019/20.
Supplementary figure 2. Counts of RSV detections from non‐sentinel sources by country in the selection of countries included, compared to the minimum (Min) and maximum (Max) detections from pre‐COVID‐19 pandemic seasons.
Supplementary figure 3. Counts (A) and percentage positivity (B) of positive specimens detected from Severe Acute Respiratory Infection (SARI) sites per country, from the selection of countries included.
Supplementary table 1: Summary of specimens tested, detections (and positivity) per country or area in the selection of countries included, surveillance system, and time period. Note: ‘No. previous seasons/inter‐seasons’ refers to the number of seasons between 2016/17 and 2019/20 that have been included in the historical data shown here. NS: ‘Not Statistically significant’.
Supplementary table 2: Summary of Moving Epidemic Method (MEM) threshold values calculated per country where possible. ‘Number of seasons included’ refers to the number of seasons included in the model run for each respective season and country combination.

## Data Availability

Data are available on request from the authors.

## References

[1] Li Y , Wang X , Broberg EK , Campbell H , Nair H , European RSV Surveillance Network . Seasonality of respiratory syncytial virus and its association with meteorological factors in 13 European countries, week 40 2010 to week 39 2019. Eurosurveillance. 2022;27(16):2100619. doi:10.2807/1560-7917.ES.2022.27.16.2100619 35451364 PMC9027150

[2] Broberg EK , Waris M , Johansen K , Snacken R , Penttinen P , European Influenza Surveillance Network . Seasonality and geographical spread of respiratory syncytial virus epidemics in 15 European countries, 2010 to 2016. Eurosurveillance. 2018;23(5):17‐00284. doi:10.2807/1560-7917.ES.2018.23.5.17-00284 PMC5801642 29409569

[3] Mook P , Meerhoff T , Olsen SJ , et al. Alternating patterns of seasonal influenza activity in the WHO European Region following the 2009 pandemic, 2010‐2018. Influenza Resp Viruses. 2020;14:150‐161, 2. doi:10.1111/irv.12703 PMC7040975 31944604

[4] Chatterjee A , Mavunda K , Krilov LR . Current state of respiratory syncytial virus disease and management. Infect Dis Ther. 2021;10(S1):5‐16. doi:10.1007/s40121-020-00387-2 33660239 PMC7928170

[5] Eden J‐S , Sikazwe C , Xie R , et al. Off‐season RSV epidemics in Australia after easing of COVID‐19 restrictions. Nat Commun. 2022;13(1):2884. doi:10.1038/s41467-022-30485-3 35610217 PMC9130497

[6] Odumade OA , van Haren SD , Angelidou A . Implications of the Severe Acute Respiratory Syndrome Coronavirus 2 (SARS‐CoV‐2) pandemic on the epidemiology of pediatric respiratory syncytial virus infection. Clin Infect Dis. 2022;75(Supplement_1):S130‐S135. doi:10.1093/cid/ciac373 35579506 PMC9129219

[7] Tang JW , Bialasiewicz S , Dwyer DE , et al. Where have all the viruses gone? Disappearance of seasonal respiratory viruses during the COVID‐19 pandemic. J Med Virol. 2021;93(7):4099‐4101. doi:10.1002/jmv.26964 33760278 PMC8250511

[8] Binns E , Koenraads M , Hristeva L , et al. Influenza and respiratory syncytial virus during the COVID‐19 pandemic: time for a new paradigm? Pediatr Pulmonol. 2022;57(1):38‐42. doi:10.1002/ppul.25719 34644459 PMC8662286

[9] Melidou A , Ködmön C , Nahapetyan K , et al. Influenza returns with a season dominated by clade 3C.2a1b.2a.2 A(H3N2) viruses, WHO European Region, 2021/22. Eurosurveillance. 2022;27(15):27. doi:10.2807/1560-7917.ES.2022.27.15.2200255 PMC9012087 35426364

[10] van Summeren J , Meijer A , Aspelund G , et al. Low levels of respiratory syncytial virus activity in Europe during the 2020/21 season: what can we expect in the coming summer and autumn/winter? Eurosurveillance. 2021;26(29):26. doi:10.2807/1560-7917.ES.2021.26.29.2100639 PMC8299745 34296672

[11] Vos LM , Teirlinck AC , Lozano JE , et al. Use of the moving epidemic method (MEM) to assess national surveillance data for respiratory syncytial virus (RSV) in the Netherlands, 2005 to 2017. Eurosurveillance. 2019;24(20):1800469. doi:10.2807/1560-7917.ES.2019.24.20.1800469 31115311 PMC6530251

[12] R Core Team . R: A language and environment for statistical computing. R Foundation for Statistical Computing, Vienna, Austria. 2022. https://www.R-project.org/

[13] Staadegaard L , Caini S , Wangchuk S , et al. The global epidemiology of RSV in community and hospitalized care: findings from 15 countries. Open forum. Open Forum Infect Dis. 2021;8(7):ofab159. doi:10.1093/ofid/ofab159 34337092 PMC8320297

[14] Lambert L , Sagfors AM , Openshaw PJM , Culley FJ . Immunity to RSV in early‐life. Front Immunol. 2014;5:5. doi:10.3389/fimmu.2014.00466 25324843 PMC4179512

[15] Di Mattia G , Nenna R , Mancino E , et al. During the COVID‐19 pandemic where has respiratory syncytial virus gone? Pediatr Pulmonol. 2021;56(10):3106‐3109. doi:10.1002/ppul.25582 34273135 PMC8441855

[16] Li Y , Wang X , Msosa T , de Wit F , Murdock J , Nair H . The impact of the 2009 influenza pandemic on the seasonality of human respiratory syncytial virus: a systematic analysis. Influenza Resp Viruses. 2021;15(6):804‐812. doi:10.1111/irv.12884 PMC8542946 34219389

[17] Bardsley M , Morbey RA , Hughes HE , et al. Epidemiology of respiratory syncytial virus in children younger than 5 years in England during the COVID‐19 pandemic, measured by laboratory, clinical, and syndromic surveillance: a retrospective observational study. Lancet Infect Dis. 2023;23(1):56‐66. doi:10.1016/S1473-3099(22)00525-4 36063828 PMC9762748

