# Supplementary material for: Seasonal and inter‐seasonal RSV activity in the European Region during the COVID‐19 pandemic from autumn 2020 to summer 2022
Source: Influenza Other Respir Viruses. 2023 Nov 20;17(11):e13219. doi: 10.1111/irv.13219 (PMC10661829; doi:10.1111/irv.13219)
Supplement: Supplementary file 1 — Figure S1: Counts (A) and percentage positivity (B) of RSV‐positive specimens detected from sentinel sources by country and season, from the selection of countries included. Previous seasons range from 2016/17 to 2019/20. Supplementary figure 2. Counts of RSV detections from non‐sentinel sources by country in the selection of countries included, compared to the minimum (Min) and maximum (Max) detections from pre‐COVID‐19 pandemic seasons. Supplementary figure 3. Counts (A) and percentage positivity (B) of positive specimens detected from Severe Acute Respiratory Infection (SARI) sites per country, from the selection of countries included. Supplementary table 1: Summary of specimens tested, detections (and positivity) per country or area in the selection of countries included, surveillance system, and time period. Note: ‘No. previous seasons/inter‐seasons’ refers to the number of seasons between 2016/17 and 2019/20 that have been included in the historical data shown here. NS: ‘Not Statistically significant’. Supplementary table 2: Summary of Moving Epidemic Method (MEM) threshold values calculated per country where possible. ‘Number of seasons included’ refers to the number of seasons included in the model run for each respective season and country combination. [file IRV-17-e13219-s001.docx]

Appendix for the original article entitled: “Seasonal and inter-seasonal RSV activity in the European Region during the COVID-19 pandemic from Autumn 2020 to Summer 2022”


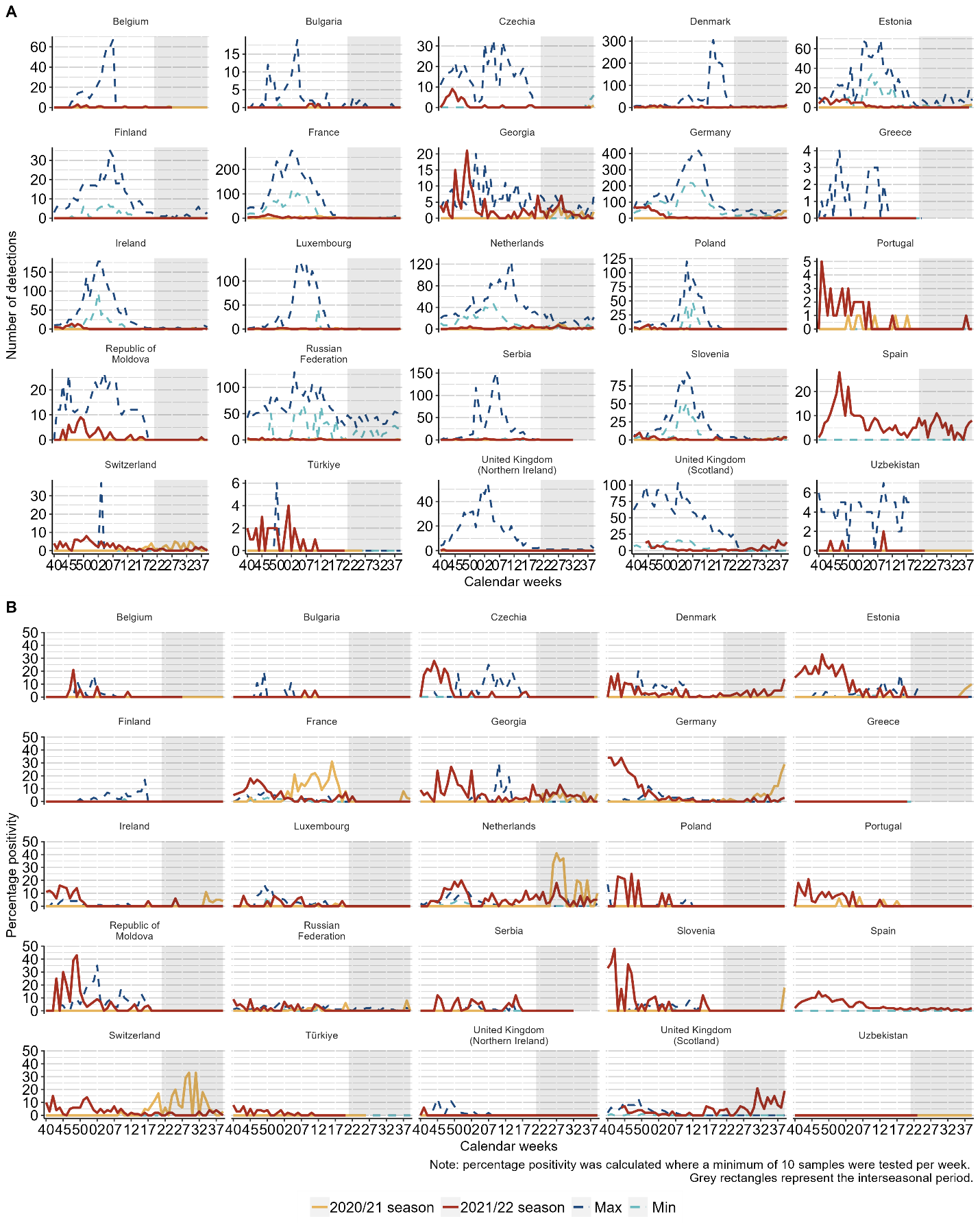


Supplementary figure 1: Counts (A) and percentage positivity (B) of RSV-positive specimens detected from sentinel sources by country and season, from the selection of countries included. Previous seasons range from 2016/17 to 2019/20.


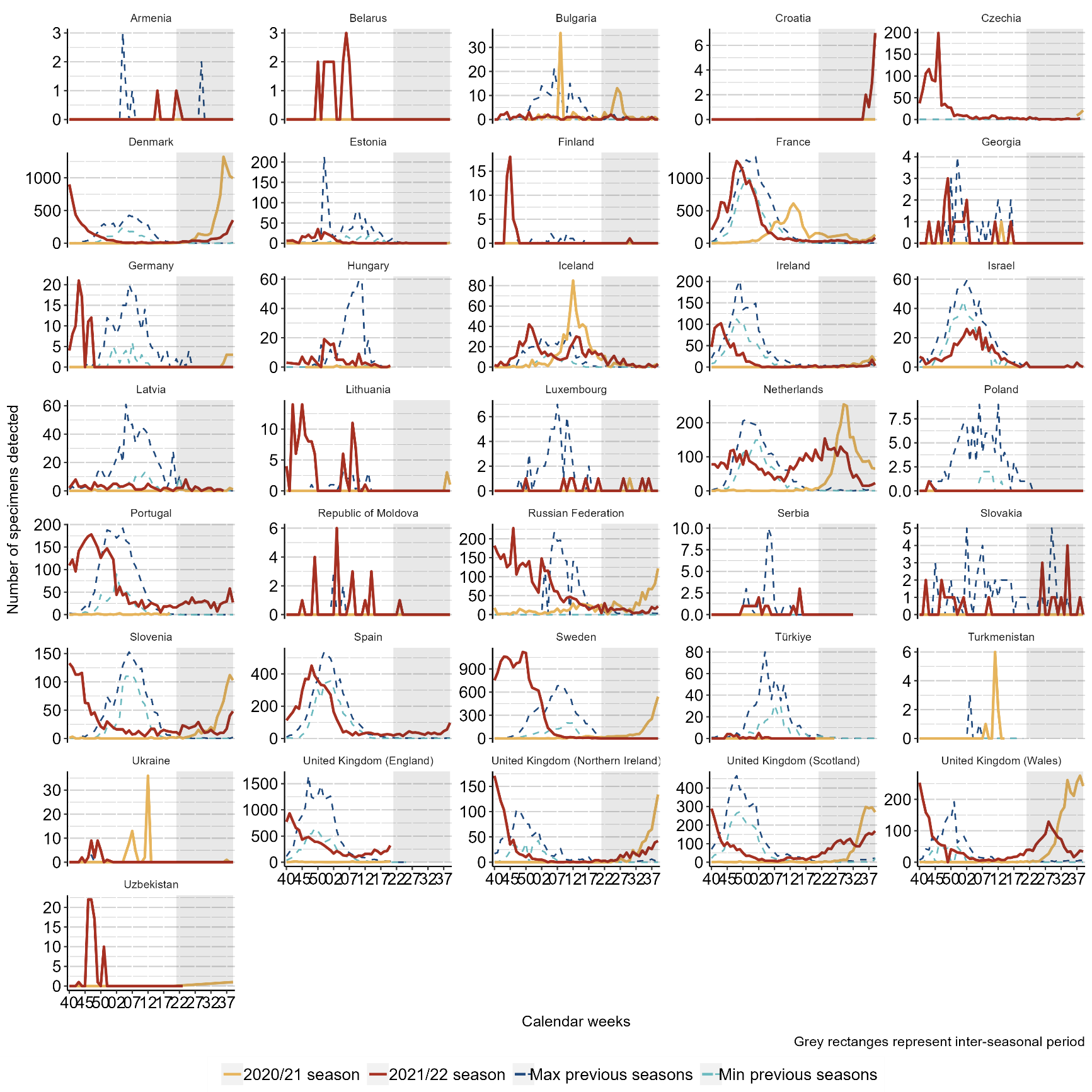


Supplementary figure 2. Counts of RSV detections from non-sentinel sources by country in the selection of countries included, compared to the minimum (Min) and maximum (Max) detections from pre-COVID-19 pandemic seasons.


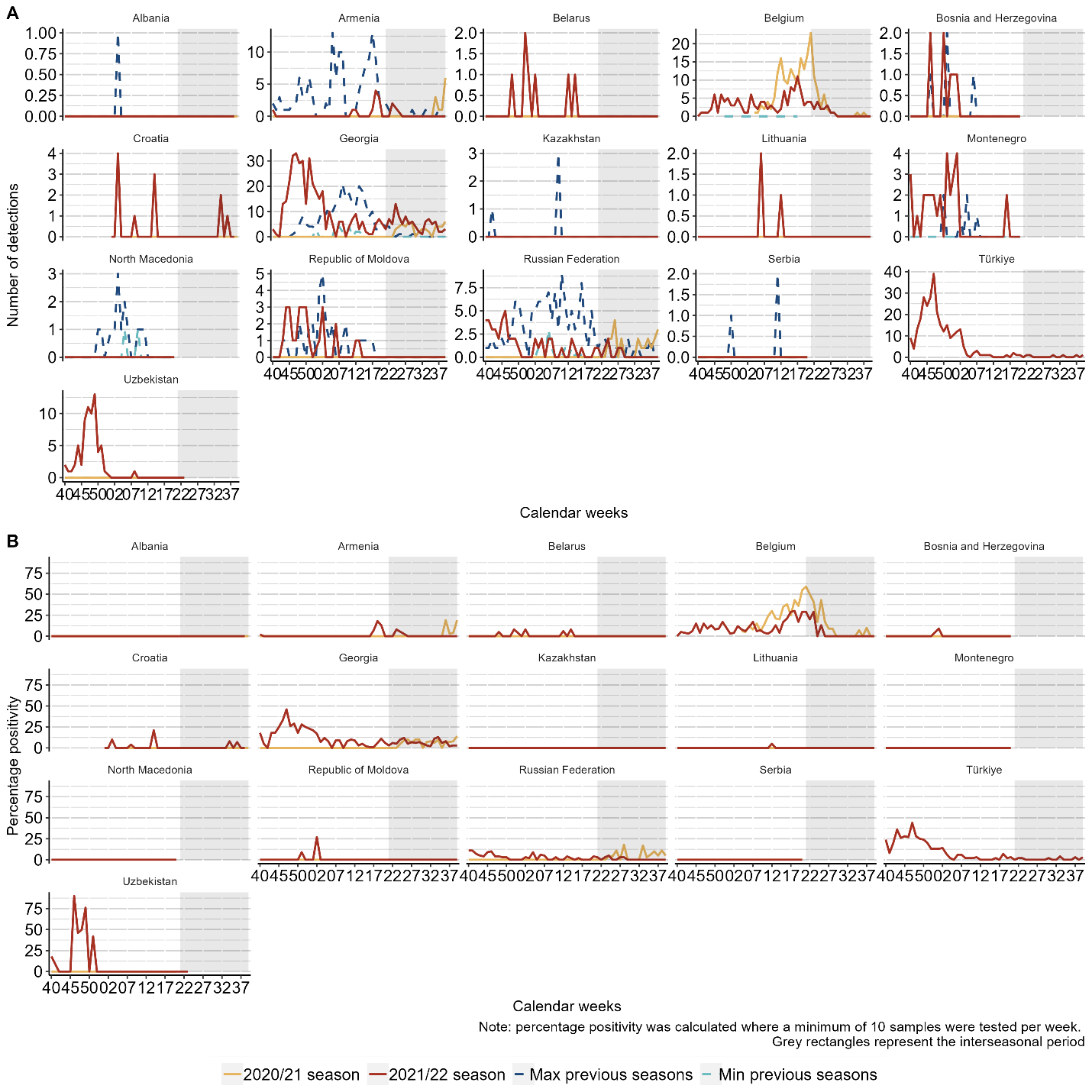


Supplementary figure 3. Counts (A) and percentage positivity (B) of positive specimens detected from Severe Acute Respiratory Infection (SARI) sites per country, from the selection of countries included.

Supplementary table 1: Summary of specimens tested, detections (and positivity) per country or area in the selection of countries included, surveillance system, and time period. Note: ‘No. previous seasons / inter-seasons’ refers to the number of seasons between 2016/17 and 2019/20 that have been included in the historical data shown here. NS: ‘Not Statistically significant’

| Country | Surveillance system | | No. previous seasons/inter-seasons | 2021/22 season | | 2020/21 season | | 2016/17-2019/20 seasons | | | |
| --- | --- | --- | --- | --- | --- | --- | --- | --- | --- | --- | --- |
|  |  |  |  | Seasonal period | Inter-seasonal period | Seasonal period | Inter-seasonal period | Seasonal period (mean) | Seasonal period (range) | Inter-seasonal period (mean) | Inter-seasonal period (range) |
| Armenia | Non-sentinel | Detections | 2 / 2 | 1 | 1 | - | - | 5 | 5 - 5 | 2 | 2 - 2 |
|  | SARI | Detections | 4 / 2 | 11 (3%) | 2 (1%) | - | 11 (5%) | 24 | 7 - 41 | 1 | 0 - 3 |
|  |  | Tests | 0 / 0 | 437 | 140 | - | 219 | - | - | - | - |
| Belarus | Sentinel | Detections | 0 / 0 | 8 (1%) | 1 (0%) | - | - | - | - | - | - |
|  |  | Tests | 0 / 0 | 542 | 487 | - | - | - | - | - | - |
|  | Non-sentinel | Detections | 0 / 0 | 15 | - | - | - | - | - | - | - |
|  | SARI | Detections | 0 / 0 | 6 (4%) | 0 (0%) | - | - | - | - | - | - |
|  |  | Tests | 0 / 0 | 159 | 76 | - | - | - | - | - | - |
| Belgium^1^ | Sentinel | Detections | 1 / 1 | 11 (3%) | 0 (0%) | - | 1 (-%) | 16 (4%) | 16 - 16 | - | - |
|  |  | Tests | 1 / - | 376 | 10 | - | 3 | 388 | 388 - 388 | - | - |
|  | SARI | Detections | 0 / 0 | 90 (9%) | 19 (18%) | 144 (24%) | 55 (25%) | - | - | - | - |
|  |  | Tests | 0 / 0 | 1,027 | 107 | 588 | 216 | - | - | - | - |
| Bosnia and Herzegovina | Sentinel | Detections | 0 / 0 | 0 (0%) | - | - | - | - | - | - | - |
|  |  | Tests | 0 / 0 | 59 | - | - | - | - | - | - | - |
|  | SARI | Detections | 1 / 0 | 6 (7%) | - | - | - | 1 | 0 - 4 | - | - |
|  |  | Tests | 0 / 0 | 82 | - | - | - | - | - | - | - |
| Bulgaria | Sentinel | Detections | 4 / 4 | 3 (2%) | - | 0 (-%) | - | 4 (11%) | 2 - 10 | 0 (0%) | 0 - 0 |
|  |  | Tests | 4 / 4 | 129 | - | 7 | - | 38 | 22 - 72 | 3 | 1 - 5 |
|  | Non-sentinel | Detections | 4 / 4 | 29 | 7 | 51 | 87 | 87 | 14 - 143 | 3 | 1 - 5 |
| Croatia^1^ | Sentinel | Detections | 0 / 0 | 0 (-%) | 9 (0%) | - | - | - | - | - | - |
|  |  | Tests | 0 / 0 | 4 | 2,275 | - | - | - | - | - | - |
|  | Non-sentinel | Detections | 0 / 0 | - | 52 | - | - | - | - | - | - |
|  | SARI | Detections | 0 / 0 | 8 (2%) | 4 (1%) | - | - | - | - | - | - |
|  |  | Tests | 0 / 0 | 471 | 372 | - | - | - | - | - | - |
| Czechia | Sentinel | Detections | 2 / 2 | 46 (6%) | 0 (0%) | - | 2 (-%) | 26 (7%) | 6 - 46 | 0 (0%) | 0 - 0 |
|  |  | Tests | 2 / 2 | 820 | 56 | - | 4 | 393 | 260 - 526 | 14 | 14 - 14 |
|  | Non-sentinel | Detections | 2 / - | 926 | 22 | - | 44 | 10 | 5 - 15 | - | - |
| Denmark | Sentinel | Detections | 1 / - | 63 (2%) | 59 (3%) | 0 (0%) | 0 (-%) | 34 (2%) | 34 - 34 | - | - |
|  |  | Tests | 1 / - | 2,641 | 1,730 | 121 | 4 | 1,751 | 1,751 - 1,751 | - | - |
|  | Non-sentinel | Detections | 4 / 4 | 3,958 | 1,493 | 8 | 6,983 | 3,935 | 2,643 - 4,489 | 17 | 1 - 40 |
| Estonia | Sentinel | Detections | 4 / 4 | 96 (14%) | 0 (0%) | 0 (0%) | 8 (5%) | 39 (7%) | 33 - 45 | 0 (0%) | 0 - 1 |
|  |  | Tests | 4 / 4 | 679 | 203 | 261 | 161 | 528 | 459 - 610 | 42 | 14 - 80 |
|  | Non-sentinel | Detections | 4 / 4 | 223 | 2 | - | 1 | 423 | 255 - 589 | 12 | 4 - 21 |
| Finland | Sentinel | Detections | 4 / 4 | 0 (0%) | 0 (0%) | 0 (0%) | 0 (-%) | 14 (6%) | 7 - 26 | 0 (0%) | 0 - 0 |
|  |  | Tests | 4 / 4 | 126 | 66 | 460 | 9 | 232 | 157 - 303 | 16 | 8 - 27 |
|  | Non-sentinel | Detections | 3 / - | 40 | 1 | - | - | 4 | 1 - 8 | - | - |
| France | Sentinel | Detections | 4 / 4 | 120 (5%) | 1 (1%) | 76 (7%) | 5 (4%) | 191 (8%) | 145 - 255 | 0 (0%) | 0 - 0 |
|  |  | Tests | 4 / 1 | 2,632 | 184 | 1,040 | 132 | 2,372 | 1,638 - 2,968 | 46 | 46 - 46 |
|  | Non-sentinel | Detections | 4 / 1 | 12,681 | 626 | 5,454 | 1,262 | 11,888 | 10,599 - 14,346 | 11 | 11 - 11 |
| Georgia | Sentinel | Detections | 4 / 4 | 122 (7%) | 33 (5%) | 1 (0%) | 21 (3%) | 6 (7%) | 0 - 11 | 0 (0%) | 0 - 1 |
|  |  | Tests | 4 / 4 | 1,687 | 728 | 1,077 | 680 | 83 | 4 - 187 | 33 | 24 - 48 |
|  | Non-sentinel | Detections | 4 / - | 14 | - | 1 | - | 6 | 3 - 11 | - | - |
|  | SARI | Detections | 4 / 2 | 355 (15%) | 98 (6%) | - | 55 (7%) | 93 | 77 - 105 | 3 | 0 - 9 |
|  |  | Tests | 4 / 2 | 2,370 | 1,630 | - | 736 | - | - | - | - |
| Germany | Sentinel | Detections | 4 / 4 | 635 (12%) | 15 (1%) | 5 (0%) | 175 (7%) | 312 (7%) | 199 - 430 | 1 (0%) | 0 - 2 |
|  |  | Tests | 4 / 4 | 5,230 | 1,624 | 4,680 | 2,477 | 4,502 | 3,803 - 5,654 | 542 | 389 - 731 |
|  | Non-sentinel | Detections | 4 / 4 | 83 | - | - | 10 | 105 | 75 - 136 | 3 | 2 - 4 |
| Greece | Sentinel | Detections | 1 / - | 0 (-%) | - | - | - | 5 (16%) | 5 - 5 | - | - |
|  |  | Tests | 1 / - | 6 | - | - | - | 32 | 32 - 32 | - | - |
| Hungary | Sentinel | Detections | 0 / 0 | 60 (15%) | - | - | - | - | - | - | - |
|  |  | Tests | 0 / 0 | 412 | - | - | - | - | - | - | - |
|  | Non-sentinel | Detections | 4 / - | 121 | - | 1 | - | 145 | 17 - 408 | - | - |
| Iceland | Non-sentinel | Detections | 4 / 4 | 488 | 72 | 503 | 44 | 246 | 216 - 276 | 2 | 2 - 3 |
| Ireland | Sentinel | Detections | 4 / 4 | 81 (5%) | 2 (1%) | 0 (0%) | 7 (2%) | 27 (3%) | 0 - 45 | 0 (0%) | 0 - 0 |
|  |  | Tests | 4 / 4 | 1,710 | 155 | 1,838 | 333 | 1,074 | 859 - 1,585 | 28 | 28 - 29 |
|  | Non-sentinel | Detections | 4 / 4 | 713 | 50 | 5 | 107 | 1,402 | 1,213 - 1,534 | 39 | 1 - 77 |
|  | SARI | Detections | 0 / 0 | 0 (0%) | 0 (0%) | - | 0 (0%) | - | - | - | - |
|  |  | Tests | 0 / 0 | 390 | 203 | - | 27 | - | - | - | - |
| Israel | Sentinel | Detections | 0 / 0 | 22 (11%) | 1 (0%) | - | - | - | - | - | - |
|  |  | Tests | 0 / 0 | 203 | 230 | - | - | - | - | - | - |
|  | Non-sentinel | Detections | 4 / - | 329 | 8 | - | - | 557 | 518 - 611 | - | - |
| Kosovo* | Sentinel | Detections | 0 / 0 | 1 (3%) | - | - | - | - | - | - | - |
|  |  | Tests | 0 / 0 | 33 | - | - | - | - | - | - | - |
| Latvia | Non-sentinel | Detections | 4 / 4 | 95 | 28 | 2 | 5 | 358 | 110 - 543 | 8 | 3 - 13 |
| Lithuania | Sentinel | Detections | 0 / 0 | 0 (-%) | 0 (-%) | 0 (-%) | - | - | - | - | - |
|  |  | Tests | 0 / 0 | 7 | 3 | 1 | - | - | - | - | - |
|  | Non-sentinel | Detections | 3 / - | 105 | - | - | 4 | 8 | 4 - 15 | - | - |
|  | SARI | Detections | 0 / 0 | 3 (11%) | - | - | - | - | - | - | - |
|  |  | Tests | 0 / 0 | 28 | - | - | - | - | - | - | - |
| Luxembourg | Sentinel | Detections | 3 / - | 13 (2%) | 2 (2%) | 6 (0%) | 0 (-%) | 53 (6%) | 36 - 68 | - | - |
|  |  | Tests | 3 / - | 773 | 131 | 4,723 | 3 | 902 | 775 - 1,092 | - | - |
|  | Non-sentinel | Detections | 3 / - | 6 | 3 | - | 1 | 22 | 2 - 42 | - | - |
| Malta | SARI | Detections | 0 / 0 | 11 (3%) | 2 (1%) | 0 (0%) | 2 (1%) | - | - | - | - |
|  |  | Tests | 0 / 0 | 412 | 305 | 94 | 135 | - | - | - | - |
| Montenegro | Sentinel | Detections | 0 / 0 | 2 (-%) | - | - | - | - | - | - | - |
|  |  | Tests | 0 / 0 | 4 | - | - | - | - | - | - | - |
|  | SARI | Detections | 2 / 0 | 27 (60%) | - | - | - | 2 | 0 - 4 | - | - |
|  |  | Tests | 0 / 0 | 45 | - | - | - | - | - | - | - |
| Netherlands | Sentinel | Detections | 4 / 4 | 57 (5%) | 40 (6%) | 0 (0%) | 39 (22%) | 84 (8%) | 64 - 104 | 1 (1%) | 0 - 1 |
|  |  | Tests | 4 / 4 | 1,106 | 690 | 415 | 180 | 1,044 | 582 - 1,493 | 167 | 78 - 321 |
|  | Non-sentinel | Detections | 4 / 4 | 2,514 | 1,441 | 42 | 2,007 | 1,891 | 1,731 - 2,020 | 24 | 10 - 32 |
| Poland | Sentinel | Detections | 4 / 4 | 23 (10%) | 0 (-%) | - | - | 14 (3%) | 6 - 26 | 0 (0%) | 0 - 0 |
|  |  | Tests | 4 / 4 | 229 | 6 | - | - | 429 | 318 - 598 | 2 | 1 - 3 |
|  | Non-sentinel | Detections | 4 / 4 | 1 | - | - | - | 44 | 26 - 77 | 2 | 1 - 2 |
| Portugal | Sentinel | Detections | 0 / 0 | 33 (6%) | 1 (3%) | 6 (2%) | - | - | - | - | - |
|  |  | Tests | 0 / 0 | 549 | 32 | 394 | - | - | - | - | - |
|  | Non-sentinel | Detections | 4 / 4 | 2,488 | 506 | 27 | - | 1,368 | 665 - 2,107 | 1 | 1 - 1 |
| Republic of Moldova | Sentinel | Detections | 3 / - | 65 (6%) | 1 (1%) | - | - | 27 (10%) | 3 - 63 | - | - |
|  |  | Tests | 3 / - | 1,026 | 92 | - | - | 265 | 114 - 359 | - | - |
|  | Non-sentinel | Detections | 2 / - | 19 | 1 | - | - | 3 | 1 - 5 | - | - |
|  | SARI | Detections | 3 / 0 | 27 (12%) | 0 (0%) | - | - | 8 | 0 - 25 | - | - |
|  |  | Tests | 3 / 0 | 223 | 2 | - | - | - | - | - | - |
| Romania^1^ | SARI | Detections | 0 / 0 | 4 (4%) | - | - | - | 4 | 1 - 5 | - | - |
|  |  | Tests | 4 / 0 | 108 | - | - | - | - | - | - | - |
| Russian Federation | Sentinel | Detections | 4 / 4 | 25 (2%) | 0 (0%) | 12 (1%) | 1 (0%) | 80 (5%) | 52 - 114 | 11 (2%) | 1 - 20 |
|  |  | Tests | 4 / 4 | 1,192 | 452 | 1,817 | 405 | 1,532 | 1,293 - 1,798 | 523 | 307 - 634 |
|  | Non-sentinel | Detections | 1 / 1 | 2,910 | 228 | 377 | 673 | 1,722 | 1,722 - 1,722 | 86 | 86 - 86 |
|  | SARI | Detections | 4 / 4 | 47 (4%) | 6 (1%) | - | 22 (5%) | 62 | 46 - 73 | 3 | 2 - 3 |
|  |  | Tests | 0 / 0 | 1,334 | 727 | - | 431 | - | - | - | - |
| Serbia | Sentinel | Detections | 4 / - | 23 (4%) | 0 (0%) | 0 (-%) | - | 9 (2%) | 5 - 16 | - | - |
|  |  | Tests | 4 / - | 512 | 22 | 5 | - | 396 | 103 - 696 | - | - |
|  | Non-sentinel | Detections | 4 / - | 12 | - | - | - | 11 | 3 - 21 | - | - |
| Slovakia | Non-sentinel | Detections | 4 / 4 | 10 | 11 | - | - | 14 | 5 - 30 | 4 | 1 - 6 |
| Slovenia | Sentinel | Detections | 4 / 4 | 49 (8%) | 12 (15%) | 0 (0%) | 16 (16%) | 47 (8%) | 31 - 60 | 0 (0%) | 0 - 0 |
|  |  | Tests | 4 / 4 | 631 | 79 | 232 | 103 | 608 | 578 - 637 | 18 | 13 - 24 |
|  | Non-sentinel | Detections | 4 / 4 | 1,156 | 347 | 13 | 569 | 1,361 | 1,225 - 1,524 | 8 | 2 - 14 |
| Spain | Sentinel | Detections | 0 / 0 | 263 (5%) | 103 (1%) | - | - | - | - | - | - |
|  |  | Tests | 0 / 0 | 5,688 | 8,031 | - | - | - | - | - | - |
|  | Non-sentinel | Detections | 4 / 4 | 4,790 | 668 | - | - | 4,222 | 3,947 - 4,578 | 12 | 9 - 16 |
| Sweden | Non-sentinel | Detections | 4 / - | 14,071 | unreported | 178 | 2,101 | 5,337 | 1,987 - 7,521 | - | - |
| Switzerland^1^ | Sentinel | Detections | 1 / - | 89 (4%) | 9 (1%) | 11 (1%) | 35 (9%) | 0 (0%) | 0 - 0 | - | - |
|  |  | Tests | 1 / - | 2,338 | 851 | 1,264 | 370 | 37 | 37 - 37 | - | - |
| Türkiye | Sentinel | Detections | 0 / 0 | 26 (2%) | - | - | - | - | - | - | - |
|  |  | Tests | 0 / 0 | 1,426 | - | - | - | - | - | - | - |
|  | Non-sentinel | Detections | 4 / 4 | 22 | - | - | - | 378 | 78 - 501 | 5 | 3 - 7 |
|  | SARI | Detections | 0 / 0 | 280 (14%) | 6 (1%) | - | - | - | - | - | - |
|  |  | Tests | 0 / 0 | 2,009 | 638 | - | - | - | - | - | - |
| Turkmenistan | Sentinel | Detections | 0 / 0 | - | - | 0 (0%) | - | - | - | - | - |
|  |  | Tests | 0 / 0 | - | - | 86 | - | - | - | - | - |
|  | Non-sentinel | Detections | 1 / - | - | - | 9 | - | 4 | 4 - 4 | - | - |
| Ukraine | Sentinel | Detections | 0 / 0 | - | - | 78 (2%) | 5 (4%) | - | - | - | - |
|  |  | Tests | 0 / 0 | - | - | 3,266 | 117 | - | - | - | - |
|  | Non-sentinel | Detections | 1 / - | 30 | - | 68 | 1 | 3 | 3 - 3 | - | - |
|  | SARI | Detections | 1 / 0 | 6 (6%) | - | - | 3 (1%) | 1 | 0 - 3 | - | - |
|  |  | Tests | 0 / 0 | 98 | - | - | 219 | - | - | - | - |
| United Kingdom (England) | Non-sentinel | Detections | 4 / - | 10,506 | 37 | 178 | - | 10,067 | 6,447 - 16,456 | - | - |
| United Kingdom (N. Ireland) | Sentinel | Detections | 3 / 3 | 1 (0%) | 0 (0%) | 0 (0%) | 0 (0%) | 12 (4%) | 7 - 17 | 0 (0%) | 0 - 0 |
|  |  | Tests | 3 / 3 | 325 | 65 | 116 | 94 | 326 | 252 - 394 | 12 | 2 - 19 |
|  | Non-sentinel | Detections | 4 / 4 | 805 | 293 | - | 522 | 690 | 429 - 977 | 22 | 9 - 48 |
| United Kingdom (Scotland) | Sentinel | Detections | 4 / 4 | 75 (3%) | 86 (9%) | - | - | 65 (7%) | 18 - 145 | 0 (0%) | 0 - 0 |
|  |  | Tests | 4 / 1 | 2,846 | 979 | - | - | 898 | 250 - 2,015 | 36 | 36 - 36 |
|  | Non-sentinel | Detections | 4 / 4 | 1,756 | 1,955 | 27 | 1,849 | 3,696 | 3,183 - 4,754 | 61 | 6 - 164 |
| United Kingdom (Wales) | Sentinel | Detections | 1 / 1 | 2 (6%) | 0 (-%) | - | 1 (-%) | 5 (50%) | 5 - 5 | 0 (0%) | 0 - 0 |
|  |  | Tests | 1 / 1 | 31 | 3 | - | 1 | 10 | 10 - 10 | 3 | 3 - 3 |
|  | Non-sentinel | Detections | 4 / 4 | 1,436 | 1,024 | 22 | 2,094 | 865 | 625 - 1,126 | 28 | 3 - 57 |
| Uzbekistan | Sentinel | Detections | 2 / - | 4 (6%) | 0 (0%) | - | - | 5 (6%) | 5 - 5 | - | - |
|  |  | Tests | 2 / - | 72 | 13 | - | - | 86 | 82 - 91 | - | - |
|  | Non-sentinel | Detections | 0 / 0 | 73 | - | - | 1 | - | - | - | - |
|  | SARI | Detections | 0 / 0 | 67 (24%) | 0 (0%) | - | - | - | - | - | - |
|  |  | Tests | 0 / 0 | 285 | 38 | - | - | - | - | - | - |
| Note: percentages were calculated when at least 10 specimens were tested. ‘-‘ refers to unreported data.  ¹ Data has been updates after data collection data, as requested by country.  *This designation is without prejudice to positions on status, and is in line with UNSCR 1244 and the ICJ Opinion on the Kosovo Declaration of Independence. All references to Kosovo in this document should be understood to be in the context of the United Nations Security Council resolution 1244 (1999). | | | | | | | | | | | |

Supplementary table 2: Summary of Moving Epidemic Method (MEM) threshold values calculated per country where possible. ‘Number of seasons included’ refers to the number of seasons included in the model run for each respective season and country combination.

|  | | Mean | | | Thresholds | | | |
| --- | --- | --- | --- | --- | --- | --- | --- | --- |
| Country | **Number of seasons included** | **Start week** | **End week** | **Duration** | **Epidemic** | **Medium** | **High** | **Very high** |
| Finland | 4 | 6 | 13 | 8 | 6.03 | 7.16 | 41.14 | 86.03 |
| France | 4 | 45 | 4 | 12 | 9.89 | 14.71 | 23.74 | 29.33 |
| Germany | 4 | 50 | 12 | 15 | 4.98 | 10.81 | 22.16 | 30.44 |
| Netherlands | 4 | 45 | 4 | 11.5 | 8.65 | 16.5 | 31.68 | 42.26 |
| Poland | 4 | 4 | 9 | 6.5 | 6.83 | 3.81 | 26.02 | 56.95 |
| Russian Federation | 4 | 5 | 15 | 9 | 8.66 | 5.11 | 16.44 | 26.73 |
| Serbia | 4 | 51 | 3 | 5 | 2.59 | 4.41 | 38.46 | 93.96 |
| Slovenia | 4 | 1 | 12 | 11.5 | 7.27 | 10.21 | 24.2 | 35.45 |
| United Kingdom (Scotland) | 4 | 42 | 3 | 14 | 12.4 | 10.23 | 30.1 | 47.78 |
